# Health, Quality of Life, and Economic Impacts of Home Care Vouchers for Middle-Income Adults

**DOI:** 10.1093/geroni/igab046.1374

**Published:** 2021-12-17

**Authors:** Joanne Spetz, Laura Wagner, Jacqueline Miller, Susan Chapman, Connie Kwong

**Affiliations:** University of California, San Francisco, San Francisco, California, United States

## Abstract

The Support at Home pilot program provided financial support for home care services by middle-income adults with disabilities in San Francisco to support aging in place. This presentation reports the results of the mixed-methods evaluation of the program, which incorporated administrative records, surveys of clients and comparison group members, surveys of informal caregivers of clients, surveys of the care providers hired by clients, and focus groups with clients and with informal caregivers. Outcome measures included the Older People’s Quality of Life Questionnaire, Patient Health Questionnaire-2, an adapted Burden Scale for Family Caregivers, and self-reported falls, emergency department visits, and hospitalizations. Analyses included pre-post chi-squared and t-test comparisons and comparisons of changes between the client and comparison groups. Multivariate regression analyses were conducted to control for demographic differences between the groups. An economic analysis was conducted to learn whether changes in costs associated with medical appointments, emergency department visits, and hospitalizations were greater than the costs of the program, including both voucher and administrative costs. Results indicated statistically significant positive changes in personal stress and financial stress, but not in the composite quality of life score. There also were statistically significant reductions in attendance at medical appointments, falls, emergency department visits, and hospitalizations. The focus group data supported the findings regarding personal and financial stress, and also indicated that clients and their caregivers perceived positive quality of life benefits. The economic analysis indicated substantial cost savings from the program due to reduced use of medical services.

